# IRSS: a web-based tool for automatic layout and analysis of IRES secondary structure prediction and searching system *in silico*

**DOI:** 10.1186/1471-2105-10-160

**Published:** 2009-05-27

**Authors:** Tzong-Yuan Wu, Chi-Chun Hsieh, Jun-Jie Hong, Chung-Yung Chen, Yuh-Show Tsai

**Affiliations:** 1 Department of Bioscience Technology, Chung Yuan Christian University, Chung-Li, 320, Taiwan; 2 Department of Biomedical Engineering, Chung Yuan Christian University, Chung-Li, 320, Taiwan; 3 R&D Center of Membrane Technology, Chung Yuan Christian University, Chung-Li, 320, Taiwan

## Abstract

**Background:**

Internal ribosomal entry sites (IRESs) provide alternative, cap-independent translation initiation sites in eukaryotic cells. IRES elements are important factors in viral genomes and are also useful tools for bi-cistronic expression vectors. Most existing RNA structure prediction programs are unable to deal with IRES elements.

**Results:**

We designed an IRES search system, named IRSS, to obtain better results for IRES prediction. RNA secondary structure prediction and comparison software programs were implemented to construct our two-stage strategy for the IRSS. Two software programs formed the backbone of IRSS: the RNAL fold program, used to predict local RNA secondary structures by minimum free energy method; and the RNA Align program, used to compare predicted structures. After complete viral genome database search, the IRSS have low error rate and up to 72.3% sensitivity in appropriated parameters.

**Conclusion:**

IRSS is freely available at this website . In addition, all source codes, precompiled binaries, examples and documentations are downloadable for local execution. This new search approach for IRES elements will provide a useful research tool on IRES related studies.

## Background

Initiation of protein translation in eukaryotes is governed by a cap- and 5' end-dependent mechanism, the scanning model, or can be mediated by a cap- and 5' end-independent manner through an RNA element termed as "internal ribosomal entry site" (IRES) [1]. The translational scanning machine, comprising the 40S ribosomal subunit and a cap-binding initiation factor complex (eIF4F, composed of eIF4E, eIF4G, and eIF4A), recognizes and binds to the 5' end methylated cap structure of mRNA and scans linearly downstream until it reaches an AUG codon embedded in an optimum context for the initiation of protein translation initiation [2]. For most eukaryotic mRNAs, the first AUG encountered by the translation initiation complex acts as the initiation codon. This is termed as the cardinal rule or the first AUG rule. In contrast to the scanning model, IRES can form specific secondary and tertiary structures and interact directly with the translational machinery beyond the AUG start codon.

IRES elements were first discovered in the mRNAs of the virus family Picornaviridae [3], which have a long highly structured 5'UTR that lacks a methylated cap structure at the 5' end. And most of the picornaviruses express a protease that specifically cleaves the eIF4G that cause the cap-binding protein eIF4E cannot assemble with the 43S ternary complex (comprising eIF3 and the 40S ribosomal subunit charged with eIF2-GTP-Met-tRNA). Thus, upon infection by the picornaviruses, host cellular protein synthesis is shut down and the viral genome is translated from IRES without competition with cellular mRNA. The cleaved eIF4G (named p100) is able to interact with the picornavirus IRESs in the absence of the eIF4E binding domain [4]. Therefore, the IRES maybe a virulence factor and the identification of IRES element of pathogenic viruses can be a benefit for the treatment of the viruses infected disease. In addition, the IRES can be employed in the development of bi-cistronic expression vector that is an important tool for the biotechnology [5]. Thus, to develop an IRES search system (IRSS) for prediction and identification of IRES element(s) in a virus genome is an important issue.

Based on the predicted secondary structure and their activity *in vitro*, the IRES elements of picornavirus are divided into four classes: type I, type II, hepatitis A virus (HAV) IRES and hepatitis C virus (HCV)-like IRES [6,7]. Type I IRES is from the enterovirus and rhinovirus genomes which are inefficient in driving translation initiation in the rabbit reticulocyte lysate (RRL) [8,9]. HeLa cells extracts are required for their optimal activity in the RRL *in vitro *translation system. In contrast, type II IRES which was found in cardioviruse and aphthoviruse genomes can initiate translation efficiently in RRL [10,11]. And the HAV IRES can also function in the RRL system [6,12]. However, the activity of the HAV IRES in the RRL *in vitro *translation system is stimulated by the liver cell extracts but not by the HeLa cells extracts [13]. HCV-like picornavirus IRES was found in Porcine teschovirus and Simian picornavirus which display IRES activity within the RRL *in vitro *translation system [14,15].

The IRES elements of the same class might have conserved primary sequence because of the functional contraction. Unfortunately, the lower homology between different IRES classes will cause inaccuracy of prediction by BLAST using primary sequences. The RNA structure prediction will therefore be useful to enhance the accuracy of de novo secondary structure prediction of IRES elements which depends somehow on good fortune. Many RNA structure prediction models have been used in RNA structure simulation, but there is no suitable model to predict the IRES element. To set up an IRES search system (IRSS), two RNA structure prediction models: comparative sequence analysis and minimum free energy structure, were applied in our IRSS. Comparative sequence analysis [16] is the gold standard for prediction of RNA secondary structure without an all-atom model. Over 97% accuracy of base pairs in ribosomal RNA secondary structures, predicted by comparative sequence analysis, were also demonstrated in high-resolution crystal structures [17-19]. However, comparative sequence analysis requires a large number of homologous sequences in database. In the absence of necessary homologous sequences, minimum free energy structure prediction can be used to predict the structure of a single RNA sequence with an average of 73% accuracy [20]. This accuracy is sufficient to serve as a starting point to build an alignment for comparative sequence analysis [21-23].

The predicted minimum free energy (MFE) structure assumes that the secondary structure is at equilibrium and provides a good simulation for the secondary structure [24]. But, thermodynamic parameters of MFE for evaluating conformation free energies are assumed without error. However, IRES element secondary structure prediction is more complicated than other RNA structures due to three different IRES types that are all related with eIF4 and 40S ribosome subunit but diverse RNA structures. In order to develop an IRES search system, we combined the MFE and RNA alignment modeling programs and adjusted the parameters to create a useful search platform for IRES prediction. To develop the IRES search system, it will be necessary to screen the database of virus sequences by the prediction of secondary structure to identify the candidate IRES element in the virus genome, especially those positive strand viruses with 5' untranslated regions. The applications of IRSS will assist biologists to either predict or discover the new viral IRES elements.

## Results

### Evaluation of IRES structure search system by Genome scanning

**Figure 1:**
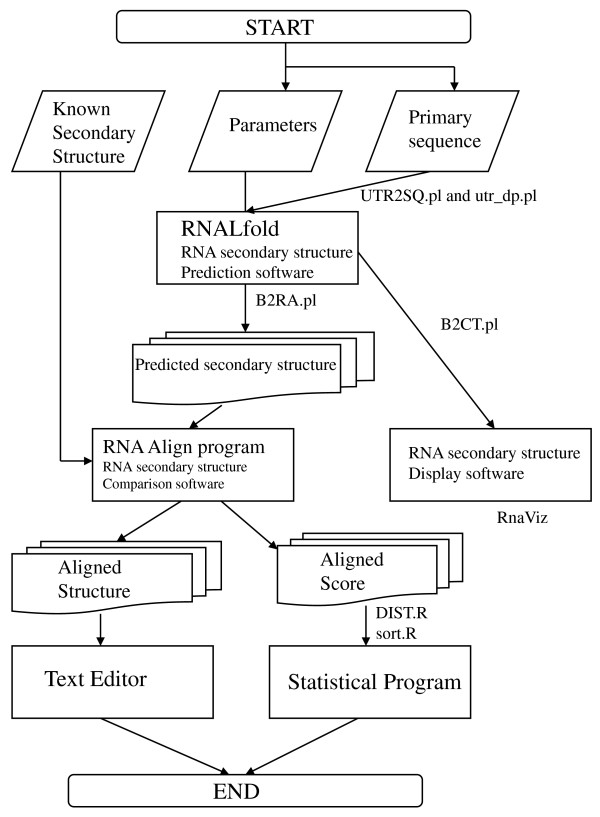
**Flowchart of IRES search system**. The RNA primary sequence is input from "START. RNA secondary structure is predicted by different L parameters and then compared with known IRES structure. The alignment score indicats the possibility of de novo IRES. The IRES structures are displayed by RnaViz software and alignment can be edited by any text editor. The six private programs are pointed beside arrow symbol and listed in Additional file 2, Additional file 3, Additional file 4, Additional file 5, Additional file 6 and Additional file 7.

**Figure 2:**
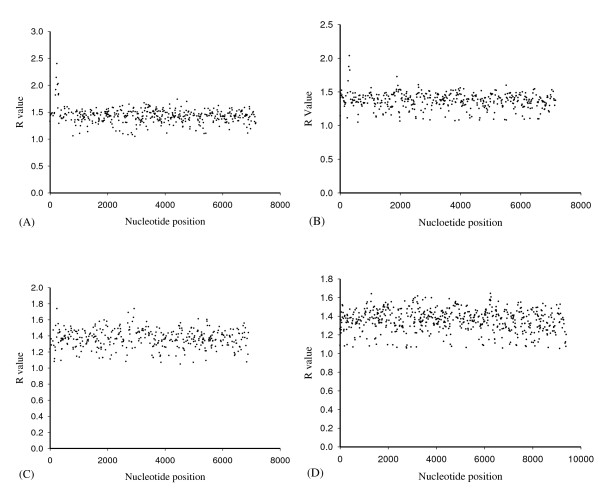
**Four virus genome sequences containing IRES element were tested in IRSS**. In window = 250 nucleotides, R value of each window was calculated as shown in (A) EV71, (B) BEV, (C) HRV and (D) HCV.

To estimate the accuracy of prediction about IRES elements by IRSS, a known IRES structure and four viral genome sequences were implanted into IRSS. Enterovirus IRES domain IV [25], was first selected as a target to compare with the whole genome sequences from four different viruses (Enterovirus 71 (U22521), Bovine Enterovirus (NC_001859), Human Rhinovirus (NC_001617) and Hepatitis C virus (NC_004102) [26-28]) were downloaded into IRSS and ran UTR2SQ.pl program to proceed RNA secondary structure prediction (see Figure 1). Those four viruses were chosen because of distantly separated evolution relationships. Both U22521 (EV71) and NC_001859 (BEV) belong to enterovirus family but of different species. Human Rhinovirus belongs to class Picornaviridae which is with the same order of enterovirus in taxonomy but of different families. Hepatitis C virus belongs to class Flaviviridae. Using our IRSS to search those four virus genomes (see Figure 2A, B, C and Figure 2D), the EV71 domain IV has been successfully predicted as an IRES structure in nucleotide position 240–444, which matches the prediction from Zell and Stelzner [29]. In this test, the L parameter of RNALfold was set to 250 (250 bases) to fetch the possible IRES structures because the length of EV71 domain IV is 205 bases.

For whole genome searching of EV71, RNALfold predicts the possible IRES structure and shown in Figure 2A. The R value presents a score for match length (ALEN) divided distance score (DIST, Y axis). The R value in position 242–444 bases has a significant higher score than the other positions (see Figure 2A). The average R value for this predicted IRES domain is 2.4 and whole genome of EV71 is 1.43 while the standard deviation is 0.14. In BEV, three predicted IRES structures around position 315 which are higher than 1.8, and position 315–549 is up to 2.03 (see Figure 2B). These predicted IRES structures located at 5'UTR site that is the appropriate region for potential IRES elements. Base 1895–2137 has also higher R value (1.72) although there are no previous reports to describe any IRES structure in this region. Theoretically, it can be either a potential IRES element or might be caused by RNA cross structure without IRES ability. The calculated average R value of BEV genome is 1.37 (SD = 0.12).

Another enterovirus, Human Rhinovirus (HRV), was applied in our IRES system to test its discriminative ability. The known HRV IRES structure is located at 5' UTR 1–618 bases. After prediction, two higher R value regions (1.74 and 1.69) at nucleotide 243–476 and nucleotide 2928–3158 as shown in Figure 2C. The second region has no experimental data to prove as an IRES structure, therefore, it may be a false positive result. The average R value is 1.36 and SD is 0.11. The last test sample is HCV, which has a different IRES type to EV71. HCV IRES is located on 5' UTR 1–340 nucleotides. From Figure 2D, no significant R value is higher than the average R value which indicates that IRSS cannot seek IRES structure precisely when the RNA Align software was adopted to compare different types of IRES elements in EV71 and HCV. For HCV, the average R value is 1.35 (SD = 0.12). To summarize the results of four viruses, matched IRES structures have been calculated to show R value over 1.7. The ambiguous range between 1.6 and 1.7 will be a potential candidate positions for IRES structure subject to more fine IRES examination.

### Linear discriminant analysis of R value and IRES element prediction from virus databases

**Figure 3:**
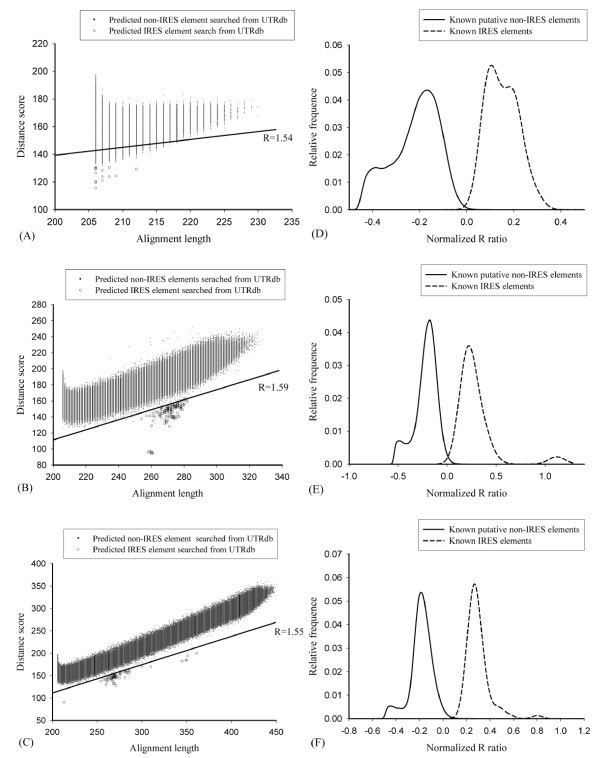
**The IRSS searching results from entire UTRdb in different length parameters**. There are three length parameters applied in this study, L = 100 (A and D), 250 (B and E) and 400 (C and F). To decide the best R value, linear discriminant analysis was applied in each window shown as d, e and f. The best R values are 1.54, 159 and 1.55 for L = 100, 250 and 400 respectively after calculation. Curves of positive and negative IRES elements represent the R value distribution. The original alignment score of every alignment window for all UTRdb data shown as (A), (B) and (C).

The second stage has two purposes. First, the IRSS search capability is evaluated while virus genomes sequences were substituted for the entire UTRdb. The known IRES element which was used for RNA comparison was selected such as HCV IRES domain III structure for example. Second, because of the diversity of known IRES length, the different length parameter (L) of RNALfold should be tested. Three L parameters, 100, 250 and 400, were applied to inspect the discrimination for IRES elements from UTRdb. To determine the best cut-off values of R value in different L parameters, the HCV and Pestivirus 5' UTR are designed as the positive group and others are the negative group. Those records were calculated their R value and estimated their distribution by linear discriminant analysis (see Figure 3D, E, F and additional file 1). The normalized R value indicates that two separate groups were made when the cut-off value was determined.

In L = 100, the R values of all virus of UTRdb were plotted in Figure 3A. To determine an appropriate cut-off value, the distributions of discriminant scores for those two groups are located at R = 1.54 (see Figure 3A and Figure 3D). Based on this cutting line, the number size of negative group is 266,192 (square dot symbol, Figure 3A) and positive group (circle symbol, potential IRES elements) contains 17 records that belong to HCV or Pestivirus (circle symbols located at R>1.73), which contains a possible IRES structure or a false positive. The predictive IRES structures were scored between R = 1.54 and 1.75, and the matched length was around 205 to 210 nucleotides. To summarize the results of R values above 1.54, the top fifteen scores were all HCV, moreover, the related virus Flaviviridae also have sixteen records that score higher than 1.54 (data not shown).

L = 250, R = 1.59 was determined as the boundary line after analysis. Circle symbols are positive controls which are the HCV IRES elements with R values greater than 1.73. The negative group (square dot symbol, Figure 3B) has 247,255 records and positive group (circle symbol, Figure 3B) has 129 records. Figure 3E manifests those two groups in linear discriminant analysis and confirms most potential IRES elements distributed below R = 1.59 (see Figure 3B and Figure 3E). In addition, there are some predicted IRES elements between R = 1.59 and R = 2.00 separated from major belt area in 240–280 matched length (X axis). The largest matched size is 328 nucleotides. Part of results matched with known IRES structures including HCV, Bovine viral diarrhea virus (BVDV), Pestivirus, Classical swine fever virus (CSFV), Border disease virus (BDV) and Hepatitis GB virus. Those are all ssRNA positive-strand viruses in class Flaviviridae. Other viruses, like Picornavirus, Enterovirus, Coronavirus, Sarcoma virus and HIV, were also found to have R values higher than 1.73 (see Table 1) and might have potential IRES structures in 5'UTR predicted from our search system.

**Table 1:** Top records of IRSS predicted potential IRES elements from UTR database in different L* parameters (without Flaviviridae)

L	Accession No	Position	SLEN^§^	Ratio	Description
100	J02342	185	101	1.57	Rous sarcoma virus
100	X51863	419	101	1.57	Avian sarcoma virus
100	AF447398	207	98	1.56	Peanut clump virus
100	AB032469	3640	97	1.55	Hop latent virus
100	AB085612	3607	95	1.55	Cucumber yellows virus
100	AJ295949	3567	101	1.55	Zucchini green mottle mosaic virus
100	AY101611	5863	99	1.55	Visna virus
100	U56902	2350	96	1.55	Citrus tristeza virus
100	X85215	332	98	1.55	Carnation Italian ringspot virus
100	Z54206	1195	100	1.55	Bovine herpesvirus 1
100	AJ011933	5064	100	1.54	Tobacco mosaic virus
250	AY064719	433	223	1.85	Simian picornavirus 12
250	AY064715	434	223	1.79	Simian picornavirus 3
250	AF406813	191	250	1.78	Porcine enterovirus 8
250	AY064708	452	223	1.78	Simian picornavirus 1
250	AF304460	13311	234	1.77	Human coronavirus 229E
250	AY064720	440	220	1.77	Simian picornavirus 15
250	U07131	1622	223	1.77	Feline calicivirus
250	D10652	1060	214	1.75	Rous sarcoma virus
250	AF026278	3154	219	1.74	Grapevine Rupestris stem pitting associated virus
250	AY008714	7423	195	1.74	Human immunodeficiency virus 1
400	Y18368	831	192	1.76	Citrus tristeza virus
400	AY008718	7412	195	1.74	Human immunodeficiency virus 1
400	X78602	1517	183	1.73	Peanut clump virus
400	AF037268	11241	198	1.72	Grapevine leafroll-associated virus 3
400	AF017149	6668	197	1.71	Hendra virus
400	AB015146	1112	227	1.70	Cucumber green mottle mosaic virus
400	AF201929	938	168	1.70	Murine hepatitis virus strain 2
400	AF280799	5143	197	1.70	Mumps virus
400	AY150312	5533	189	1.70	Porcine reproductive and respiratory syndrome virus
400	AY274119	11849	194	1.70	SARS coronavirus Tor2
400	D10371	4863	194	1.70	Phocine distemper virus
400	L04998	684	295	1.70	Human herpesvirus 5

*length of sequence fragments

^§^sequence length

The discriminant R value is 1.55, L = 400 and the frequency of group R ratio is shown in Figure 3C and Figure 3F. There are 235,554 records in negative group (square dot symbol, Figure 3C) and 3,862 data in positive group (circle symbol, Figure 3C). The largest matched length is 452 nucleotides. In Figure 3C, the positive group located between R = 1.55 to 2.00 in alignment length between 250 and 290 contains 69 records of HCV and Pestivirus. The higher L value seems to filter out lots of candidate IRES structures; beside Flaviviridae, only five other virus predicted IRES found in top ten records (see Table 1). Two of them, Citrus tristeza virus and Human immunodeficiency virus 1(HIV1), have the same as results in L = 250 but in different predicted positions.

The comparison of all positive groups in L = 100, 250 and 400 might reveal false positive and wrong prediction of IRES structures. The distributions of two groups from different L values are matched our goal to predict "HCV IRES element" but results are obviously diverse after IRSS search (see Table 1, Figure 3A, B, and Figure 3C). The IRES structure prediction ability adopted by our search design is confirmed.

### Accuracy of IRSS

To evaluate accuracy rate of the IRES prediction system, two known IRES elements, HCV IRES domain III and IRES of Pestivirus, and entire UTR database were analyzed in IRSS. However, from BLAST and RNA comparison results (data not shown), the primary and second structures of Pestivirus IRES are similar to HCV IRES domain III which might be attributed to the same Flaviviridae order. For RNA Align software, both Pestivirus IRES and HCV IRES were selected as the standard IRESes for IRSS. The UTR database version 19 contains 39 sequences of HCV 5'UTR and 113 sequences of Pestivirus IRES, which were counted known as IRES elements to examine the accuracy of IRSS. From Figure 3D, E and Figure 3F, discriminant R values are 1.54, 1.59 and 1.55 in L parameters as 100, 250 and 400 respectively. After estimation, sensitivity was calculated in different L lengths and better sensitivity was found in L = 250. For HCV IRES standard, the sensitivity score was 66.7% but the accuracy of Pestivirus IRES prediction was up to 72.3% in L = 250.

### Web-based tools

**Figure 4:**
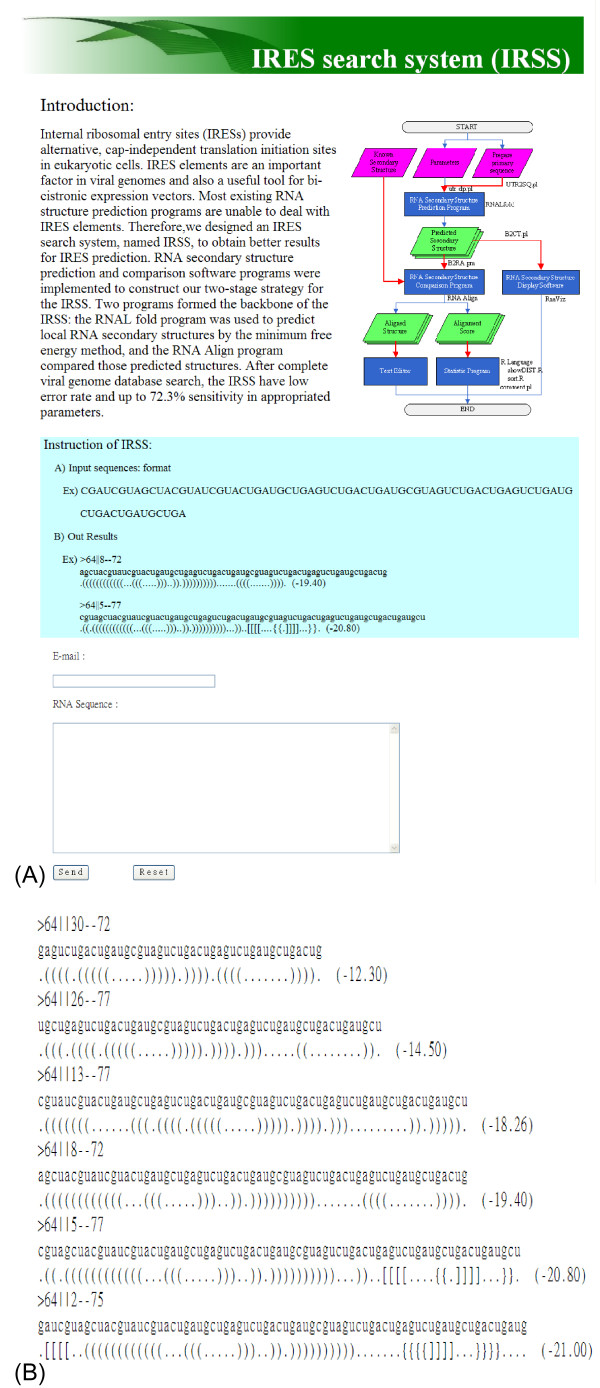
**Web-based IRES search system**. (A) Input window, sequences as FASTA or plain text formats. (B) The example of output data. All of the results with R scores higher than 1.5 after IRSS prediction can be listed. Those data include potential IRES sequences, potential secondary structures and predicted minimum free energies.

The IRSS tool is available in web-based on line search as . The original RNA prediction software and perl-script programs, such as RNAL fold, RNA Align, UTR2SQ.pl...etc., have been transferred into Web service style and executed automatically. Figure 4 showed the input window and output example. The input sequences can be FASTA and/or plain text formats and results are in pain text which is able to be read by any word processing software. The default L parameter of web-based IRSS is 250 and R value is 1.4. The IRSS web tool is run in a Linux workstation which has Fedora 6.0 operation system.

## Discussion

IRESes have been applied as biotechnological tools, particularly for gene expression. Functional and mutational studies have also been demonstrated on different IRES structure analysis [30,31]. Can a scientist predict the potential IRES elements before experiment? There are lots of software to predict the RNA secondary structures but there is no available software to predict the IRES elements. Recently, experimentally verified IRES database has been built in [32]. This database collects the full-length sequences of all mRNAs manifesting IRES activity. A similar work to collect experimentally verified IRES data has been done as UTR database which was also applied in our study. To set up the IRES search tool, we modified and combined two software to become a search flow. The RNAL fold is based on minimum free energy method, thus, longer sequences will reduce its accuracy. That is the major reason why L = 250 is better than L = 400. Minimum free energy prediction has been adopted by most of RNA secondary structure prediction software, unfortunately, its sensitivity is about 72% [20]. This explains that the sensitivity of our IRES search system is less than 72.3%. To conquer this problem, separated predictions for IRES different type structures might be the better option. However, occurrence of more false positives and longer computer CPU running time are the disadvantages. Therefore, more information is required to rule out false positives. The second software, RNA Align program, can compare first and secondary structures of RNA for a precise specific prediction of conserved structures such as Hairpin loop, Budge loop and Interior loop. On the other hand, RNA Align cannot hasten its calculation unless it is replaced by other programs or modifying the source codes.

**Figure 5:**
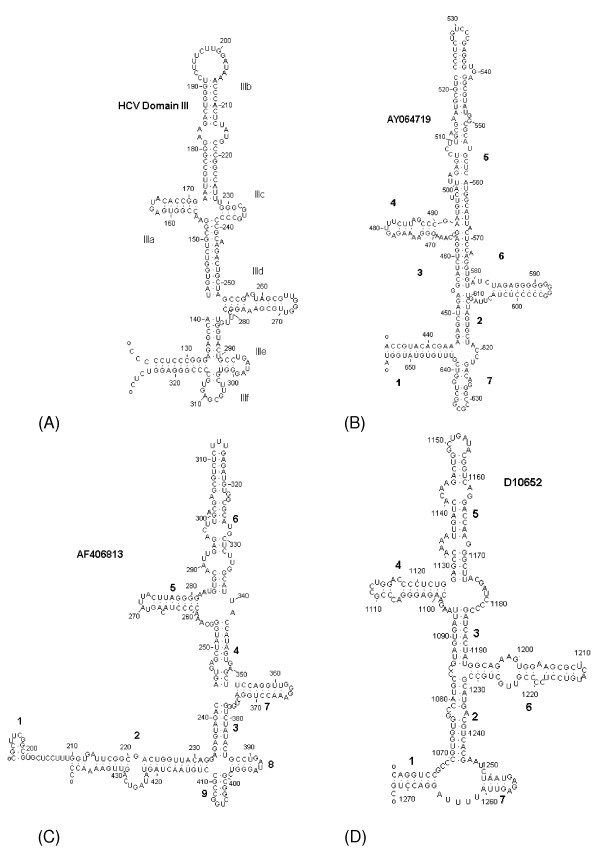
**Prediction IRES structures of Simian picornavirus, PEV-8 and RSV**. (A) HCV Domain III. (B) Predicted IRES structure of Simian picornavirus in nucleotide position 433–655 (L = 250). (C) Predicted IRES structure of PEV-8 in nucleotide position 191–440 (L = 250). (D) Predicted IRES structure of RSV in nucleotide position 1060–1273 (L = 250).

Based on results obtained from IRSS, HCV and Pestivirus are major members of positive group in different L parameters. However, positive group may contain other virus which might be potential IRES elements. For example, Simian picornavirus has high R value (1.85) in 430–655 nucleotides. Comparison of the two predicted RNA secondary structures, Simian picornavirus domains 2, 4 and 6 (see Figure 5B) are similar to HCV domain IIIf, IIIa and IIId (see Figure 5A) respectively. This IRES element has been proved by Chard [33]. Similarities between the two whole structures is up to 41.7% which results to high R value region in Simian picornavirus by IRSS. Another ssRNA positive strand virus, Porcine enterovirus 8, has been discovered as potential IRES element at 190 nucleotides in L = 250 (see Figure 5C). Its secondary structure also contains domains similar to HCV domains IIIa, IIIb, IIId and IIIf, that was recently proved by Pisarev [15]. The IRES elements of Simian picornavirus and Porcine enterovirus 8 genomes which matched our search results of IRSS. Moreover, IRSS can distinguish known IRES elements from UTRdb (see Figure 3D, E, and Figure 3F). Our results demonstrate that IRSS is not only to predict RNA secondary structures but also to locate the IRES elements. However, in L = 100 and 400, none of Porcine enterovirus showed potential IRES structure due to various predicted structures in different L parameters, but two Retroviridae viruses, Rous Sarcoma virus and HIV, were located at positive group in our calculation. Rous Sarcoma virus occupies four top scores in L = 100 and one of them, D10652, appears in L = 250 too (see Figure 5D). The R value of 1060–1272 nucleotide within D10652 is 1.75 (cutting line R = 1.59, L = 250), which is sequence inside gag polyprotein coding region. Thus, this record shows to be false positive. Another Rous Sarcoma virus regions, (X51863 and M21526), were found to have IRES element at 5'UTR. This element forms a few stem loops similar to HCV domains IIIa, IIIb, IIId and IIIe (data not shown). But this IRES element conformation is smaller than HCV and Simian picornavirus. More experimental evidences are necessary to prove this IRES element.

Pattern searching program and web service have been developed such as Rfam from Sanger institute [34]. Rfam is a collection of multiple RNA sequence alignments using covariance models to represent consensus primary sequences of non-coding RNA families. Rfam will provide information not only IRESes but also other RNAs. In contrast to Rfam, IRSS is specific for IRES study. IRSS searches IRES elements by structure comparison that contains neighbor regions for structure prediction and avoids short consensus primary sequences problem to improve IRES structures prediction. However, IRSS requires verified IRES structures to improve accuracy of RNA Align program and is different approach to sequence alignment of Rfam.

From the initial test for our IRES search system, Enterovirus 71 and related virus were successful to find the IRES element but failed to apply in other virus families. Species specificity is indeed an important factor in this test. During the second test, longer RNA sequences might cause difficulty in prediction for RNAL program which resulted to less positive results in L = 400. When L parameter was 100, shorter predicted length was easy to locate sub-structure that caused lots of predicted IRES elements that were focused in the same area and also revealed more false positive results. Predicted sub-domains of IRES element might match one of individual HCV domains resulting to the loss of the ability to fetch whole IRES element. After evaluation of all length parameters, middle size (L = 250) of prediction can cover whole IRES structure and also avoids the disadvantages of minimum free energy method. In addition, to improve sensitivity of IRSS, we are also preparing the implementation of a new designed program which will allow us to do similar interactions between 40s rRNA and IRES domains by 3D model. Furthermore, we plan to provide our IRES search system with a web-based interface which will help to define IRES elements. Finally, we believe that the IRSS will provide a useful source for IRES location before experimental study. The IRSS can be a public resource. It can facilitate the scientific community not only to analyze using IRSS as a tool, but also a means of communication through provide feedbacks.

## Conclusion

We report the new IRES search system (IRSS), which is a search flowchart to facilitate IRES elements' prediction and analysis. The dicistronic test for IRES elements verification is the gold standard despite of the inefficiency in experiments which have serious translation background problems and lack of appropriate prediction software. In addition, there are many RNA structure prediction models, but there is no suitable model to predict IRES elements. To achieve this purpose, IRSS combined "minimum free energy structure prediction" program and "comparative sequence analysis" program. The accuracy of IRSS is sufficient to serve as a starting point and to provide bioinformatic evidences for IRES element experiment and application. Finally, IRSS has not only been developed as a useful system for prediction of IRES elements but also been transformed as a web-based service to allow public usage.

## Methods

### Methodology of IRES element prediction

Two key steps are the backbone of the IRES elements search system (IRSS): 1) RNA folding and 2) comparison. RNAL fold program [35] is the first step and functions to predict the RNA secondary structure by minimum free energy method. The second step is RNA secondary structure comparison which matches the known IRES structures executed by RNA Align program [36]. In our designed IRSS, primary RNA sequences inputted into search flowchart (see Figure 1) with individual length parameter (L) is transferred as raw RNA sequences into RNAL fold input format by "UTR2SQ.pl" [see Additional file 2] and the "utr_dp.pl" program [see Additional file 3]. The utr_dp.pl is the major control batch program to link each stage of IRSS. The output data of RNAL fold is then transformed into RNA Align format by B2RA.pl program [see Additional file 4]. The results of RNA Align software will be displayed as two files: Aligned structure and Alignment score files. Two statistical programs, DIST.R and sort.R [see Additional file 5 and Additional file 6], analyze those alignment scores and calculate the score distribution [37]. For RNA view, B2CT.pl [see Additional file 7] changes the predicted RNA secondary structure into "connect file format" (*.ct) which will read by RnaViz [38] to display in screen and print. The essential prerequisite step of this analysis is the calculation of secondary structure's stability by folding sub-sequences of length, L.

The length of sequence (L) fragments is assigned for window sizes, which the window slides along the target sequences. The length size is a varied factor in IRES prediction. In this study, the L parameter of RNAL fold program was set as L = 400, 250 or 100. All three length parameters input into our IRSS can predict all possible RNA secondary structures. The algorithms of RNA Align software consider all RNA structures that can be cataloged as base-match, base-mismatch, base-deletion, arc-match, arc-breaking, arc-altering and arc-removed [36]. Each RNA alignment is measured through the similarities between two RNA structure sequences as the 'edit distance', which aims on the calculation of transforming/editing one sequence into the other. Nucleotide insertion, deletion and substitution are three transforming types of edit distance. The score of the alignment between RNA structures is dependent to the summation of costs that were computed by 'edit distance' [36].

The known IRES elements were selected as standard structures for our IRSS. In "RNA family database of alignments and common motifs" (Rfam, ) [34,39], the known RNA structures including IRES are qualified in our system. There are twelve IRES models built upon consensus sequences in Rfam database. Those models are based on the similar consensus secondary sequences that are predicted by PFOLD program [40]. Moreover, those IRES consensus secondary sequences are the major templates for RNA alignment software such as RNAL fold program. In IRSS, if RNAL fold program predicted IRES elements that cannot match IRES models of Rfam or fetch at least two homolog IRESs from related species, those input data will be discarded.

### Practice of the IRES element search system

Four different whole virus sequences, EV71, BEV, HRV and HCV, were tested in IRES search system. All coding sequences were downloaded from GenBank  with accession number, U22521, NC_001859, NC_001617 and NC_004102[26-28]. The purpose of this test is to look for EV71 IRES domain IV (240–444 nucleotides) from those virus sequences using our IRES search system. Furthermore, in order to understand the precision of IRSS, the entire virus 5' UTR database (UTRdb, ) and the target is HCV domain III (accession umber: AF177037) [41-43], was input into the IRSS. Domain III of the HCV IRES positions at the initiation codon in the ribosomal mRNA binding cleft by binding the 40S subunit [44].

In RNA align software, two factors are considered to evaluate the IRES elements that can be predicted by our IRSS, distance score (DIST) and alignment match length (ALEN) from RNA Align program. DIST represents the score of secondary structure in comparison with the default score of each RNA structure (base-deletion, base-mismatch, arc-mismatch, are-removing, arc-altering and arc-breaking) adopted in RNA align software. Because DIST value will increase concomitantly with longer alignment length, DIST score fails to specify the significance of matched structures from shorter and bigger alignment sequences. Therefore, DIST and ALEN are transformed into a ratio which is defined as R = ALEN/DIST. The R values are collected from all predicted IRES elements including known IRES and potential candidate IRES elements. Linear discriminant analysis (LDA) analyzes all R values to make a discriminant line that distinguishes candidate IRES group and non-IRES group. The error rate of IRES search system is estimated in comparison of known IRES structures with candidate IRES elements.

## Authors' contributions

C-CH carried out the software design, and performed all of the IRSS task. C-YC participated in the statistical analysis, drafted the manuscript, drew figures and submitted manuscript as the correspondence author. T-YW launched this study, and participated in its design and coordination and helped to draft the manuscript. Y-ST participated in the design of the study. All authors read and approved the final manuscript.

## Supplementary Material

Additional file 1 **Statistical analysis of length parameters in IRSS**. A table shows the statistic results for linear discriminant analysis (LDA) in different length parameters. Click here for file

Additional file 2 **Program perl script: UTR2SQ.pl**. A perl source code represents the program to transfer the sequences from UTR database into a temporary file. Click here for file

Additional file 3 **Program perl script: utr_dp.pl**. A perl source code represents the program to transfer the temporary file from UTR2SQ.pl into RNAL fold input format. Click here for file

Additional file 4 **Program perl script: B2RA.pl**. A perl source code represents the program to transfers the output data of RNAL fold into RNA Align format. Click here for file

Additional file 5 **Program R script: DIST.R**. R source code represents the program to analyze all alignment scores and calculate the score distribution. Click here for file

Additional file 6 **Program R script: sort.R**. R source code represents the program to transform the output data from DIST.R into a table format which can be read by Microsoft^® ^Excel^® ^program. Click here for file

Additional file 7 **Program perl script: B2CT.pl**. A perl source code represents the program to reformats the predicted RNA secondary structure into "connect file format" (*.ct). Click here for file

## References

[B1] Pelletier J, Sonenberg N (1988). Internal initiation of translation of eukaryotic mRNA directed by a sequence derived from poliovirus RNA. Nature.

[B2] Dever TE (1999). Translation initiation: adept at adapting. Trends Biochem Sci.

[B3] Jang SK, Pestova TV, Hellen CU, Witherell GW, Wimmer E (1990). Cap-independent translation of picornavirus RNAs: structure and function of the internal ribosomal entry site. Enzyme.

[B4] Ochs K, Rust RC, Niepmann M (1999). Translation initiation factor eIF4B interacts with a picornavirus internal ribosome entry site in both 48S and 80S initiation complexes independently of initiator AUG location. J Virol.

[B5] Finkelstein Y, Faktor O, Elroy-Stein O, Levi BZ (1999). The use of bi-cistronic transfer vectors for the baculovirus expression system. J Biotechnol.

[B6] Belsham GJ (2009). Divergent picornavirus IRES elements. Virus Res.

[B7] Fernandez-Miragall O, Lopez de Quinto S, Martinez-Salas E (2009). Relevance of RNA structure for the activity of picornavirus IRES elements. Virus Res.

[B8] Alexander L, Lu HH, Wimmer E (1994). Polioviruses containing picornavirus type 1 and/or type 2 internal ribosomal entry site elements: genetic hybrids and the expression of a foreign gene. Proc Natl Acad Sci USA.

[B9] Honda M, Ping LH, Rijnbrand RC, Amphlett E, Clarke B, Rowlands D, Lemon SM (1996). Structural requirements for initiation of translation by internal ribosome entry within genome-length hepatitis C virus RNA. Virology.

[B10] Brown BA, Ehrenfeld E (1979). Translation of poliovirus RNA in vitro: changes in cleavage pattern and initiation sites by ribosomal salt wash. Virology.

[B11] Dorner AJ, Semler BL, Jackson RJ, Hanecak R, Duprey E, Wimmer E (1984). In vitro translation of poliovirus RNA: utilization of internal initiation sites in reticulocyte lysate. J Virol.

[B12] Borman AM, Kean KM (1997). Intact eukaryotic initiation factor 4G is required for hepatitis A virus internal initiation of translation. Virology.

[B13] Glass MJ, Jia XY, Summers DF (1993). Identification of the hepatitis A virus internal ribosome entry site: in vivo and in vitro analysis of bicistronic RNAs containing the HAV 5' noncoding region. Virology.

[B14] Chard LS, Bordeleau ME, Pelletier J, Tanaka J, Belsham GJ (2006). Hepatitis C virus-related internal ribosome entry sites are found in multiple genera of the family Picornaviridae. J Gen Virol.

[B15] Pisarev AV, Chard LS, Kaku Y, Johns HL, Shatsky IN, Belsham GJ (2004). Functional and structural similarities between the internal ribosome entry sites of hepatitis C virus and porcine teschovirus, a picornavirus. J Virol.

[B16] Gutell RR, Lee JC, Cannone JJ (2002). The accuracy of ribosomal RNA comparative structure models. Curr Opin Struct Biol.

[B17] Ban N, Nissen P, Hansen J, Moore PB, Steitz TA (2000). The complete atomic structure of the large ribosomal subunit at 2.4 A resolution. Science.

[B18] Schluenzen F, Tocilj A, Zarivach R, Harms J, Gluehmann M, Janell D, Bashan A, Bartels H, Agmon I, Franceschi F (2000). Structure of functionally activated small ribosomal subunit at 3.3 angstroms resolution. Cell.

[B19] Wimberly BT, Brodersen DE, Clemons WM, Morgan-Warren RJ, Carter AP, Vonrhein C, Hartsch T, Ramakrishnan V (2000). Structure of the 30S ribosomal subunit. Nature.

[B20] Mathews DH (2004). Using an RNA secondary structure partition function to determine confidence in base pairs predicted by free energy minimization. Rna.

[B21] Diamond JM, Turner DH, Mathews DH (2001). Thermodynamics of three-way multibranch loops in RNA. Biochemistry.

[B22] Flamm C, Hofacker IL, Maurer-Stroh S, Stadler PF, Zehl M (2001). Design of multistable RNA molecules. Rna.

[B23] Mathews DH, Banerjee AR, Luan DD, Eickbush TH, Turner DH (1997). Secondary structure model of the RNA recognized by the reverse transcriptase from the R2 retrotransposable element. Rna.

[B24] Clote P, Ferre F, Kranakis E, Krizanc D (2005). Structural RNA has lower folding energy than random RNA of the same dinucleotide frequency. Rna.

[B25] Thompson SR, Sarnow P (2003). Enterovirus 71 contains a type I IRES element that functions when eukaryotic initiation factor eIF4G is cleaved. Virology.

[B26] Brown BA, Pallansch MA (1995). Complete nucleotide sequence of enterovirus 71 is distinct from poliovirus. Virus Res.

[B27] Earle JA, Skuce RA, Fleming CS, Hoey EM, Martin SJ (1988). The complete nucleotide sequence of a bovine enterovirus. J Gen Virol.

[B28] Kolykhalov AA, Agapov EV, Blight KJ, Mihalik K, Feinstone SM, Rice CM (1997). Transmission of hepatitis C by intrahepatic inoculation with transcribed RNA. Science.

[B29] Zell R, Stelzner A (1997). Application of genome sequence information to the classification of bovine enteroviruses: the importance of 5'- and 3'-nontranslated regions. Virus Res.

[B30] Tang S, Collier AJ, Elliott RM (1999). Alterations to both the primary and predicted secondary structure of stem-loop IIIc of the hepatitis C virus 1b 5' untranslated region (5'UTR) lead to mutants severely defective in translation which cannot be complemented in trans by the wild-type 5'UTR sequence. J Virol.

[B31] Varaklioti A, Georgopoulou U, Kakkanas A, Psaridi L, Serwe M, Caselmann WH, Mavromara P (1998). Mutational analysis of two unstructured domains of the 5' untranslated region of HCV RNA. Biochem Biophys Res Commun.

[B32] Mokrejs M, Vopalensky V, Kolenaty O, Masek T, Feketova Z, Sekyrova P, Skaloudova B, Kriz V, Pospisek M (2006). IRESite: the database of experimentally verified IRES structures. Nucleic Acids Res.

[B33] Chard LS, Kaku Y, Jones B, Nayak A, Belsham GJ (2006). Functional analyses of RNA structures shared between the internal ribosome entry sites of hepatitis C virus and the picornavirus porcine teschovirus 1 Talfan. J Virol.

[B34] Griffiths-Jones S, Moxon S, Marshall M, Khanna A, Eddy SR, Bateman A (2005). Rfam: annotating non-coding RNAs in complete genomes. Nucleic Acids Res.

[B35] Hofacker IL, Priwitzer B, Stadler PF (2004). Prediction of locally stable RNA secondary structures for genome-wide surveys. Bioinformatics.

[B36] Jiang T, Lin G, Ma B, Zhang K (2002). A general edit distance between RNA structures. J Comput Biol.

[B37] Tuplin A, Evans DJ, Simmonds P (2004). Detailed mapping of RNA secondary structures in core and NS5B-encoding region sequences of hepatitis C virus by RNase cleavage and novel bioinformatic prediction methods. J Gen Virol.

[B38] De Rijk P, Wuyts J, De Wachter R (2003). RnaViz 2: an improved representation of RNA secondary structure. Bioinformatics.

[B39] Griffiths-Jones S, Bateman A, Marshall M, Khanna A, Eddy SR (2003). Rfam: an RNA family database. Nucleic Acids Res.

[B40] Knudsen B, Hein J (2003). Pfold: RNA secondary structure prediction using stochastic context-free grammars. Nucleic Acids Res.

[B41] Pesole G, Liuni S, Grillo G, Ippedico M, Larizza A, Makalowski W, Saccone C (1999). UTRdb: a specialized database of 5' and 3' untranslated regions of eukaryotic mRNAs. Nucleic Acids Res.

[B42] Pesole G, Liuni S, Grillo G, Saccone C (1998). UTRdb: a specialized database of 5'- and 3'-untranslated regions of eukaryotic mRNAs. Nucleic Acids Res.

[B43] Yanagi M, Purcell RH, Emerson SU, Bukh J (1999). Hepatitis C virus: an infectious molecular clone of a second major genotype (2a) and lack of viability of intertypic 1a and 2a chimeras. Virology.

[B44] Boehringer D, Thermann R, Ostareck-Lederer A, Lewis JD, Stark H (2005). Structure of the hepatitis C Virus IRES bound to the human 80S ribosome: remodeling of the HCV IRES. Structure (Camb).

