# Supplementary material for: IRSS: a web-based tool for automatic layout and analysis of IRES secondary structure prediction and searching system in silico
Source: BMC Bioinformatics. 2009 May 27;10:160. doi: 10.1186/1471-2105-10-160 (PMC2698906; doi:10.1186/1471-2105-10-160)
Supplement: Additional file 1 — Statistical analysis of length parameters in IRSS. A table shows the statistic results for linear discriminant analysis (LDA) in different length parameters. [file 1471-2105-10-160-S1.pdf]

Additional file 1: Statistic analysis data of virus genome search by IRSS in different L parameters

	**L=100**				**L=250**				**L=400**			
	Group Negative		Group Positive		Group Negative		Group Positive		Group Negative		Group Positive	
N	266192		17		247255		129		235554		69	
	ALEN	DIST	ALEN	DIST	ALEN	DIST	ALEN	DIST	ALEN	DIST	ALEN	DIST
Min	206.0	130.5	206.0	115.5	206.0	127.3	254.0	94.5	206.0	127.8	213.0	90.75
1^{st}QU	206.0	154.3	206.0	121.0	211.0	162.0	268.0	141.0	232.0	175.8	266.0	144.5
Median	207.0	161.0	206.0	126.5	244.0	177.5	272.0	148.5	317.0	226.5	270.0	148.0
Mean	208.2	163.4	207.1	125.5	246.5	179.3	271.1	145.4	312.7	230.6	275.8	150.3
3rdQU	210.0	170.3	208.0	129.8	277.0	194.8	275.0	153.0	387.0	284.8	280.0	155.5
MAX	230.0	197.5	212.0	130.5	328.0	252.3	282.0	1618	452.0	360.5	361.0	200.25
SD	3.2	12.4	1.6	4.8	33.2	21.3	5.7	13.0	77.9	58.0	24.7	15.6
VAR1	10.2	154.7	2.6	22.8	1104.6	451.8	32.9	169.7	606.8	3364.3	609.9	242.35
VAR2	11.14		2.0		560.1		48.5		4388.2		351.0	
