# Supplementary material for: IRSS: a web-based tool for automatic layout and analysis of IRES secondary structure prediction and searching system in silico
Source: BMC Bioinformatics. 2009 May 27;10:160. doi: 10.1186/1471-2105-10-160 (PMC2698906; doi:10.1186/1471-2105-10-160)
Supplement: Additional file 2 — Program perl script: UTR2SQ.pl. A perl source code represents the program to transfer the sequences from UTR database into a temporary file. [file 1471-2105-10-160-S2.pdf]

Additional file 2: UTR2SQ.pl

```
#!/usr/bin/perl

use strict;
use warnings;

use File::Basename;
use lib dirname __FILE__;
use CCHLOG;

if ($#ARGV >= 0) { # 0 means 1 argument
    unless (-e $ARGV[0]) {
        print "Config file \'$ARGV[0]\' not found! Abort! $!\n";
        exit;
    }

} else {
    print 'Usage:', "\n";
    print '      UTR2SQ.pl ConfigFileName [Start [End]]', "\n";

    exit;
}

open (CFGFH, $ARGV[0]) or die "Can't open config file \'$ARGV[0]\'! $!\n";

chomp (my $datfilename = <CFGFH>);

chomp (my $outputpath = <CFGFH>);
mkdir ($outputpath, 0777) or die "Cannot mkdir $outputpath: $!";
my $total_file = "./$outputpath/total.txt";
my $BTF_file = "./$outputpath/BTF.err";

chomp (my $outputlogfilename = <CFGFH>);
my $logfilename = "./$outputpath/$outputlogfilename";

chomp (my $param = <CFGFH>);

chomp (my $call_utr_dp = <CFGFH>);
```

```
my $dpcfgfile;
if ($call_utr_dp eq 'y') {
	chomp ($dpcfgfile = <CFGFH>);
}
close (CFGFH);

# Start sequence number (default = 1)
my $start_num = 1;
if ($ARGV[1]) {
	$start_num = $ARGV[1];
}
# End sequence number (default = not define = end of file)
my $end_num;
if ($ARGV[2]) {
	$end_num = $ARGV[2];
}

open (CFGFH, $ARGV[0]);
my @totalcfg = <CFGFH>;
close (CFGFH);
vislog ($logfilename, "@ARGV\n@totalcfg\n");


#-----------------------------------------------------------------
# Start

open (FH, $datfilename) || die "Could not open $datfilename!";
vislog ($logfilename, localtime()." UTR2SQ.pl\n");

my $acc_num;
my $seq_count = 0;
my $str_count = 0;

while (my $item = <FH>) {
	my @items = split ' ', $item;

	if ($items[0] eq 'ID') {
```

```
        # new sequence
        $seq_count++;

        if (($end_num) && ($seq_count > $end_num)){
            last;
        }
    } elsif ($items[0] eq 'AC') {
        if ($seq_count >= $start_num) {
            $acc_num = $items[1];
            chomp $acc_num;
            chop $acc_num; # drop ';'

            repvislog ($logfilename,
                sprintf ("\n%10d AC: %s\n", $seq_count, $acc_num), '.');
        }
    } elsif ($items[0] eq 'SQ') {
        if ($seq_count >= $start_num) {
            invislog ($logfilename,
                sprintf ("                SQ: length: %d\n", $items[2]));

            # Get one sequence form input file.
            my $seq;
            for (my $i = 0; $i < $items[2]/60; $i++) {
                my $seq_line = <FH>;
                my @seq_line_items = split ' ', $seq_line;

                pop @seq_line_items; # drop last item (numbering)
                my $seq_one_line = join '', @seq_line_items;
                $seq .= $seq_one_line;
            }

            # Open an temp file and print the sequence into temp file
            my $tempfile = 'temp';

            opcfile ('>'.$tempfile, ">$acc_num\n$seq");

            # Structure prediction of the sequence in temp file
            system "RNALfold $param < temp > $acc_num.txt";
```

```
            unlink $tempfile;

            my $temp_count = count ("$acc_num.txt");

            # Test if structure prediction was failed for this sequence..

            if ($temp_count > 0) {
                append_file ($total_file, $acc_num.'.txt', 'AC '.$acc_num);
                append_string ($total_file, "END\n");
            }
            unlink $acc_num.'.txt';
            $str_count += $temp_count;

        } # if ($seq_count >= $start_num)
    } # elsif ($items[0] eq 'SQ')

} # while (my $item = <FH>)
close FH;

my $real_seq_count = $seq_count - $start_num;
vislog ($logfilename, "\nSequence count: $real_seq_count\n");
vislog ($logfilename, "Structure count: $str_count\n");

if ($call_utr_dp eq 'y') {
    exec "perl ../../bin/utr_dp.pl $dpcfgfile";
}

exit;

# ---------------------------------------------------------
# Program-wide subroutine

sub count {
    my $file = shift;
    open (TEMPFH, $file) || die "Could not open $file to count!";

    my $found_structures = 0;
    my $line = <TEMPFH>; # drop first line
```

```
    my $line_count = 1;

    while (<TEMPFH>) {
        $line_count++;
        chomp;

        my @line_items = split; # must be split

        if ($line_items[0] =~ m/\(/) { # search for '('
            $found_structures++;
        } elsif ($line_items[0] =~ m/[agct]/i) {
            # end if we meet ATGC... etc.
            last;
        } else {
            invislog ($logfilename,
                "Line $line_count contain no structure!\n");
        }
    }

    close TEMPFH;

    if ($line_count == 1) {
        invislog ($logfilename,
            "BTF in f3\n");
        invislog ($BTF_file, "$acc_num\n");
        unlink $acc_num.'.txt';
    }
    return $found_structures;
}


# --------------------------------------------------------
# System wide subroutine

sub opcfile {
    my $tempfile = shift;
    my $print_string = shift;
```

```
    open (TEMPFH, $tempfile) or
        die "opcfile(): Could not open $tempfile!";
    print TEMPFH $print_string;
    close TEMPFH;
}
```
