# Supplementary material for: IRSS: a web-based tool for automatic layout and analysis of IRES secondary structure prediction and searching system in silico
Source: BMC Bioinformatics. 2009 May 27;10:160. doi: 10.1186/1471-2105-10-160 (PMC2698906; doi:10.1186/1471-2105-10-160)
Supplement: Additional file 3 — Program perl script: utr_dp.pl. A perl source code represents the program to transfer the temporary file from UTR2SQ.pl into RNAL fold input format. [file 1471-2105-10-160-S3.pdf]

Additional file 3: utr_dp.pl

```
#!/usr/bin/perl -w
# --------------------------------------------------------
use strict;
use warnings;
#use File::Copy;

use File::Basename;
use lib dirname __FILE__;
use CCHLOG;
use B2RA;
print @ARGV,"\n", $#ARGV, "\n";
if ($#ARGV >= 0) { # 0 means 1 argument
    unless (-e $ARGV[0]) {
        print "Config file \'$ARGV[0]\' not found! Abort! $!\n";
        exit;
    }

} else {
    print 'Usage:', "\n";
    print '    utr_dp.pl ConfigFileName [Start [End]]', "\n";

    exit;
}

open (CFGFH, $ARGV[0]) or die "Can't open config file \'$ARGV[0]\'! $!\n";

my $outputpath = <CFGFH>; chomp $outputpath;
my $templatefile = <CFGFH>; chomp $templatefile;
unless (-e $templatefile) {
    qlog ("We need template file: \'$templatefile\'!");
    die;
}
my $call_next = <CFGFH>; chomp $call_next;
close (CFGFH);

# --------------------------------------------------------
```

```
my $starttime = time;
my $log_file = "./$outputpath/utr_dp.log";
my $total_ali = "./$outputpath/total.ali";
my $total_alilog = "./$outputpath/total.ali.log";
my $total_file = "./$outputpath/total.txt";

unlink $log_file;
unlink $total_ali;
unlink $total_alilog;

my $score_file = "./$outputpath/score.csv";
unlink $score_file;

invislog ($score_file, "AC, START_POS, STRU_LEN, DIST, ALI_LEN, RATIO\n");

my $start_num = 1;

if ($ARGV[1]) {
    $start_num = $ARGV[1];
}

my $end_num; # undefine $end_num means reach the end!
if ($ARGV[2]) {
    $end_num = $ARGV[2];
}

# --------------------------------------------------------
my $OS = check_OS();
qlog (localtime()." utr_dp $OS");

# --------------------------------------------------------
open (CFGFH, $ARGV[0]);
my @totalcfg = <CFGFH>;
close (CFGFH);
qlog ("@ARGV\n@totalcfg\n");

# --------------------------------------------------------
open (TOTAL, $total_file) || die "Can't open total.txt!!";
```

```
my $seq_count = 0;
my $stru_count = 0;

while (<TOTAL>) {
    my $AC;
    my $name;

    my @lineitems = split ' ', $_;

    # Every sequence start with AC and end with END
    # Here we cut total.txt into pieces of sequence
    if ($lineitems[0] eq 'AC') {
        $seq_count++;

        if (($end_num) && ($seq_count > $end_num)){
            last;
        }

        $AC = $lineitems[1];

        my $next_line;
        my $write_sequence;
        do {
            $next_line = <TOTAL>;

            unless ($next_line =~ m/^END/) {
                $write_sequence .= $next_line;
            }
        } until ($next_line =~ m/^END/);

        unless ($seq_count >= $start_num) {
            next;
        }

        qlog ($AC.' '.localtime().' '.$seq_count);

        # write file
```

```
    $name = $AC.'.txt';
    open (TOTALTEMP, '>'.$name) || die "Can't open $name!!: $!";
    print TOTALTEMP $write_sequence;
    close TOTALTEMP;
} else {
    qlog ("Strange Format?");
    next;
}

#--------------------------------------------------------------
# Calculate valid structures in sequence.
# Prepare structures.
open (TEMP, $name) || die "Can't open $name?";
<TEMP>; #drop first line

my @structs;
my $code;
my $found_structures = 0;
my $line_count = 1; # because we drop first line.

while (<TEMP>) {
    chomp;

    $line_count++;
    my @line_items = split; # must be split

    if ($line_items[0] =~ m/\(/) { # search for '('
        #my @out = split (//,$_);

        #print '['.$line_items[0],"]\n";
        $found_structures++;
        push @structs, $_;

    } elsif ($line_items[0] =~ m/[agct]/i) {
        # end if we meet ATGC... etc.
        $code = $_;
        last;
    } else {
```

```
            print "Line $line_count contain no structures!\n";
        }
    }
    close (TEMP);
    unlink $name;

    qlog ($found_structures.' structure(s).');

    #-----------------------------------------------------------
    # we can do b2ra and run rna_align now

    if (($code) && ($found_structures > 0)) {

        my $count = 0;

        foreach my $item (@structs) {
#                my @temp = split (" ", join ("", @{$item})); # re-split by " "
#                my @br = split("",$temp[0]);

#                my $len = $#br + 1;
#                my $code_start = $temp[$#temp]-1;
#                my $code_end = $code_start + $#br;
#                my @tempcodes = @codes[$code_start..$code_end];

            $count++;
            @_ = split ' ', $item;
            my $br = $_[0];
            my $len = length($br);
            my $code_start = $_[$#_]-1;
            my $tempcode = substr ($code, $code_start, $len);

            br2ra ($br, $tempcode, 'temp.ra');
            #B2RA::br2ct (\@br, \@tempcodes, 'temp.ct');

            $stru_count++;
            #-----------------------------------------------------------
            # Call DP
            qlog ('DP POS:'.($code_start+1).' '.$count.'/'.$found_structures.' '.$seq_count.'
```

```
'.$stru_count.' ['.localtime().']');

            my $dp_command =
                "dp $templatefile temp.ra original.param temp.ali > temp.ali.log";
            if ($OS eq 'UNIX') {
                $dp_command = "./".$dp_command;
            }
            my $ret_val = system $dp_command;

            getrascore ('temp.ali.log', $AC, $code_start+1, $len);

            append_ali ($AC, $code_start+1, $len);

            if    ($ret_val != 0) {
                qlog ('DP failed and crashed at '.localtime());
                # Although DP crashed, we still try
                #       to finish other calculations.
            }

            #------------------------------------------------------------
            #append_ra_ct ($AC, $temp[$#temp], $#br+1); # AC, Start_Pos, Length
            unlink 'temp.ra'; #unlink 'temp.ct';

        } # foreach my $item (@structs)
    } # if ((@codes) && ($found_structures > 0))



} # while (<TOTAL>)

close (TOTAL);

my $endtime = time;
my $diff_time = $endtime - $starttime;
print $diff_time, "\n";

# Use gzip and tar to pack results
pack_dir ($outputpath);
```

```
if ($call_next eq 'y') {
	if (-e "next.pl") {
		rename "next.pl", "next.pl.done";
		exec "perl ./next.pl.done";
	}
}

exit;
# ==========================================================

# -------------------------------------------------------
# Program-wide subroutine
sub qlog {
	my $log_string = shift;

	vislog ($log_file ,$log_string."\n");
}

#sub append_ra_ct {
#	my $name = shift;
#	my $start_pos = shift;
#	my $len = shift;
#
#	my $sep_string = 'AC: '.$name.' POS: '.$start_pos.' LEN: '.$len;
#
#	append_file ($total_ra_file, 'temp.ra', $sep_string);
#	append_file ($total_ct_file, 'temp.ct', $sep_string);
#}

sub append_ali {
	my $name = shift;
	my $start_pos = shift;
	my $len = shift;

	my $sep_string = 'AC: '.$name.' POS: '.$start_pos.' LEN: '.$len;

	append_file ($total_ali, 'temp.ali', $sep_string);
	append_file ($total_alilog, 'temp.ali.log', $sep_string);
```

```
}

# getrascore -----------------------------------------------------
sub getrascore {
	my $ra_log_file = shift;
	my $AC = shift;
	my $start = shift;
	my $slen = shift;

	open (RALOGFH, $ra_log_file) || die "Couldn't open $ra_log_file!";
	my $prev_line;
	my $last_line;
	while (my $line = <RALOGFH>) {
		$prev_line = $last_line;
		$last_line = $line;
	}
	close RALOGFH;

	my @last_line_items = split '=', $last_line;
	if ($last_line_items[0] eq 'length') {
		chop $prev_line;
		my $len = $last_line_items[1]/2;

		open (SCOREFH, '>>'.$score_file) || die "Couldn't open $score_file!";

		printf "$AC, %8d, score: %d, length: %d, l/s: %8f\n",
			$start, $prev_line, $len, $len/$prev_line;

		printf SCOREFH "$AC, %8d, %8d, %8f, %8d, %8f\n",
			$start, $slen, $prev_line, $len, $len/$prev_line; #prev_line=score,(distance)

		close SCOREFH;
	} else {
		print "Failed! $!";
	}
}

sub pack_dir {
```

```
    my $dir = shift;

    if (-e "./$dir/total.txt") {
        system "gzip ./$dir/total.txt";
    }

    system "gzip ./$dir/total.ali";
    system "gzip ./$dir/total.ali.log";

    system "tar -cvf $dir.tar $dir";
    system "gzip $dir.tar";
}

# --------------------------------------------------------
# System wide subroutine

sub check_OS {

    my $OS = '';

    unless ($OS) {
        unless ($OS = $^O) {
            require Config;
            $OS = $Config::Config{'osname'};
        }
    }

    if ($OS =~ /^MSWin/i) {
        $OS = 'WINDOWS';
    } elsif ($OS =~ /^VMS/i) {
        $OS = 'VMS';
    } elsif ($OS =~ /^dos/i) {
        $OS = 'DOS';
    } elsif ($OS =~ /^MacOS/i) {
        $OS = 'MACINTOSH';
    } elsif ($OS =~ /^os2/i) {
        $OS = 'OS2';
    } elsif ($OS =~ /^epoc/i) {
```

```
        $OS = 'EPOC';
    } elsif ($OS =~ /^cygwin/i) {
        $OS = 'CYGWIN';
    } else {
        $OS = 'UNIX';
    }

    return $OS;
}
```
