# Supplementary material for: IRSS: a web-based tool for automatic layout and analysis of IRES secondary structure prediction and searching system in silico
Source: BMC Bioinformatics. 2009 May 27;10:160. doi: 10.1186/1471-2105-10-160 (PMC2698906; doi:10.1186/1471-2105-10-160)
Supplement: Additional file 4 — Program perl script: B2RA.pl. A perl source code represents the program to transfers the output data of RNAL fold into RNA Align format. [file 1471-2105-10-160-S4.pdf]

Additional file 4: B2RA.pl

```
#!/usr/bin/perl -w
use strict;
use warnings;

use File::Basename;
use lib dirname __FILE__;
use B2RA;

my $name = $ARGV[0];

my $output_name = basename ($name).".ra";
if ($ARGV[1]) {
    $output_name = $ARGV[1];
}

    open (TEMP, $name) || die "Can't open $name?";
    <TEMP>; #drop first line

    my @structs;
    my $code;
    my $found_structures = 0;
    my $line_count = 1; # because we drop first line.

    while (<TEMP>) {
        chomp;

        $line_count++;
        my @line_items = split; # must be split

        if ($line_items[0] =~ m/\(/) { # search for '('
            $found_structures++;
            push @structs, $_;

        } elsif ($line_items[0] =~ m/[agct]/i) {
            # end if we meet ATGC... etc.
            $code = $_;
            last;
```

```
    } else {
        print "Line $line_count contain no structures!\n";
    }
}
close (TEMP);

if (($code) && ($found_structures > 0)) {
    my $count = 0;
    foreach my $item (@structs) {
        $count++;
        @_ = split ' ', $item;
        my $br = $_[0];
        my $len = length($br);
        my $code_start = $_[$#_]-1;
        my $tempcode = substr ($code, $code_start, $len);

        br2ra ($br, $tempcode, $output_name);
    }
}
```
