# Supplementary material for: IRSS: a web-based tool for automatic layout and analysis of IRES secondary structure prediction and searching system in silico
Source: BMC Bioinformatics. 2009 May 27;10:160. doi: 10.1186/1471-2105-10-160 (PMC2698906; doi:10.1186/1471-2105-10-160)
Supplement: Additional file 5 — Program R script: DIST.R. R source code represents the program to analyze all alignment scores and calculate the score distribution. [file 1471-2105-10-160-S5.pdf]

Additional file 5: DIST.R

```
#
test <- read.csv("1-.csv", col.names=c("ac", "startpos", "slen","score", "alen", "ratio"))
attach(test)

# Low level plot
# Ratio
plot.new();
plot.window(xlim=c(1,2.75), ylim=c(0,6))
axis (1);axis (2);box();
lines (density(ratio,bw=0.001))
title (xlab="Ratio", ylab="Count");
rug(ratio)

# DIST (score)
plot.new();
plot.window(xlim=c(90,250), ylim=c(0,0.01))
axis (1);axis (2);box();
lines (density(score,bw=0.5))
title (xlab="Distance score", ylab="Count");
rug(score)

plot (density(score,bw=0.25), xlim=c(90,250))

# ALEN
plot.new();
axis (1);axis (2);box();
plot (density(alen,bw=0.5), xlim=c(200,320))

rug(alen)

# SLEN
plot.new();
axis (1);axis (2);box();
plot (density(slen,bw=0.5), xlim=c(0,250))

rug(slen)
```
