# Supplementary material for: IRSS: a web-based tool for automatic layout and analysis of IRES secondary structure prediction and searching system in silico
Source: BMC Bioinformatics. 2009 May 27;10:160. doi: 10.1186/1471-2105-10-160 (PMC2698906; doi:10.1186/1471-2105-10-160)
Supplement: Additional file 6 — Program R script: sort.R. R source code represents the program to transform the output data from DIST.R into a table format which can be read by Microsoft® Excel® program. [file 1471-2105-10-160-S6.pdf]

Additional file 6: sort.R

```
test <- read.csv("1-.csv", col.names=c("ac", "startpos", "slen","score", "alen", "ratio"))
ratio <- sort (test$ratio, decreasing=TRUE, index.return=TRUE)
out <- data.frame(ac=test$ac[ratio$ix], startpos=test$startpos[ratio$ix],
slen=test$slen[ratio$ix], score=test$score[ratio$ix], alen=test$alen[ratio$ix],
ratio=test$ratio[ratio$ix])
write.table(out, file = "1-.sorted.csv", sep = ",")
```
